# Quantitative evaluation of taste dysfunction using electrogustometry in patients with head and neck cancer undergoing intensity-modulated radiation therapy and intensity-modulated proton therapy

**DOI:** 10.1093/jrr/rrag074

**Published:** 2026-09-21

**Authors:** Tomoka Shima, Koichi Yasuda, Masaaki Suzuka, Shigeru Yamano, Akiko Takeuchi, Nanami Iwai, Yuta Kanno, Yasuhiro Dekura, Hideki Minatogawa, Yoshihiro Fujita, Tomohiko Miyazaki, Fuki Koizumi, Shuhei Takahashi, Hidefumi Aoyama, Kazuyuki Minowa

**Affiliations:** Department of Radiology, School of Dental Medicine and Graduate School of Dental Medicine, Hokkaido University, Kita 13, Nishi 7, Kita-ku, Sapporo, 060-8586, Japan; Department of Radiation Oncology, Hokkaido University Hospital, Kita 14, Nishi 5, Kita-ku, Sapporo, 060-8648, Japan; Department of Radiology, School of Dental Medicine and Graduate School of Dental Medicine, Hokkaido University, Kita 13, Nishi 7, Kita-ku, Sapporo, 060-8586, Japan; Department of Radiology, School of Dental Medicine and Graduate School of Dental Medicine, Hokkaido University, Kita 13, Nishi 7, Kita-ku, Sapporo, 060-8586, Japan; Department of Radiology, School of Dental Medicine and Graduate School of Dental Medicine, Hokkaido University, Kita 13, Nishi 7, Kita-ku, Sapporo, 060-8586, Japan; Center for Cause of Death Investigation, Graduate School of Medicine, Hokkaido University, Kita 15, Nishi 7, Kita-ku, Sapporo, 060-8638, Japan; Department of Radiology, School of Dental Medicine and Graduate School of Dental Medicine, Hokkaido University, Kita 13, Nishi 7, Kita-ku, Sapporo, 060-8586, Japan; Department of Radiology, School of Dental Medicine and Graduate School of Dental Medicine, Hokkaido University, Kita 13, Nishi 7, Kita-ku, Sapporo, 060-8586, Japan; Department of Radiation Oncology, Hokkaido University Hospital, Kita 14, Nishi 5, Kita-ku, Sapporo, 060-8648, Japan; Department of Radiation Oncology, Hokkaido University Hospital, Kita 14, Nishi 5, Kita-ku, Sapporo, 060-8648, Japan; Department of Radiation Oncology, Hokkaido University Hospital, Kita 14, Nishi 5, Kita-ku, Sapporo, 060-8648, Japan; Department of Radiation Oncology, Hokkaido University Hospital, Kita 14, Nishi 5, Kita-ku, Sapporo, 060-8648, Japan; Department of Radiation Oncology, Faculty of Medicine, Hokkaido University, Kita 15, Nishi 7, Kita-ku, Sapporo, 060-8638, Japan; Department of Radiation Oncology, Hokkaido University Hospital, Kita 14, Nishi 5, Kita-ku, Sapporo, 060-8648, Japan; Department of Radiation Oncology, Faculty of Medicine, Hokkaido University, Kita 15, Nishi 7, Kita-ku, Sapporo, 060-8638, Japan; Department of Radiology, School of Dental Medicine and Graduate School of Dental Medicine, Hokkaido University, Kita 13, Nishi 7, Kita-ku, Sapporo, 060-8586, Japan

**Keywords:** taste dysfunction, electrogustometry, head and neck cancer, intensity-modulated radiation therapy, intensity-modulated proton therapy

## Abstract

Taste disorders are common adverse events in patients undergoing radiotherapy (RT) for head and neck cancer (HNC), significantly impairing quality of life. This study was performed to quantitatively evaluate taste function using electrogustometry (EGM) in patients treated with intensity-modulated radiation therapy (IMRT) or intensity-modulated proton therapy (IMPT). This retrospective study included 18 patients with HNC (15 men, 3 women; median age 61 years) treated with IMRT or IMPT. EGM and patient-reported taste questionnaires were assessed at baseline, completion of RT and 3, 6, and 12 months post-treatment. EGM thresholds were measured bilaterally at tongue sites using standardized protocols. All patients completed EGM assessments, while questionnaire data were partially missing at treatment completion. Longitudinal changes in objective and subjective taste function were analyzed to evaluate radiation-induced taste dysfunction and recovery patterns over 1 year. Patient-reported taste scores showed significant longitudinal changes across five time points (*P* < 0.001), worsening at completion of RT and gradually recovering thereafter. EGM thresholds also changed significantly over time (*P* < 0.001), although no significant effects of treatment group or interaction were observed. No significant differences between IMRT and IMPT groups were found for changes from baseline except at 12 months (*P* = 0.04). In this exploratory study, EGM demonstrated significant time-dependent changes in taste function after RT for HNC, with no significant differences between IMRT and IMPT in the primary analysis. IMPT showed a trend toward milder dysfunction, but findings are preliminary and require confirmation in larger prospective studies.

## INTRODUCTION

Standard treatment for head and neck cancer (HNC) includes surgery, chemotherapy and radiation therapy, either alone or in combination. Radiation therapy for HNC can cause a range of adverse events, including oral mucositis, xerostomia and taste disorders [1–5]. These adverse events not only worsen nutritional status and reduce quality of life (QOL), but they also make it difficult to continue treatment and may even affect life expectancy. Therefore, it is important to assess and manage oral adverse events to ensure completion of radiation therapy and maintain the patients' QOL.

Taste typically begins to decline approximately 3–4 weeks after the start of radiotherapy (RT) and gradually recovers during the first few months following treatment [2, 6, 7]. However, in some patients, taste loss persists and continues to interfere with QOL even after 1 year [6]. Taste assessment methods used in cancer treatment include patient-reported outcome measures [8, 9], clinician-reported measures such as the Common Terminology Criteria for Adverse Events (CTCAE) [10, 11], and objective measures using liquid tastants or filter paper disks/strips [2, 12, 13]. Taste is a complex, integrated sensation involving multiple senses. Conventional testing with taste solutions has limitations; quantitative measurement is difficult because of diffusion of the solution and variability in stimulus intensity, and the procedure itself is time-consuming. By contrast, electrogustometry (EGM), which evaluates gustatory nerve function by measuring the electric taste produced when the tongue is stimulated by an electric current, allows for easy quantitative measurement [14, 15]. EGM can stimulate a small, localized area and significantly shortens the testing time.

The use of intensity-modulated radiation therapy (IMRT) with X-rays or intensity-modulated proton therapy (IMPT) with proton beams in recent years has enabled radiation oncologists to deliver effective doses to gross tumors and areas at risk for microscopic disease [5, 11, 16]. IMRT and IMPT are believed to achieve tumor control while minimizing radiation exposure to organs-at-risk (OAR), such as the salivary glands, oral cavity and facial muscles, thereby reducing adverse events including taste disturbances [17]. For patients with HNC undergoing IMRT or IMPT, it will be increasingly important in future clinical practice to quantitatively assess taste disturbances and compare adverse events with those seen in conventional RT. To the best of our knowledge, no studies have yet investigated the use of EGM in head and neck RT patients treated with IMRT or IMPT over an extended period, including both before and after RT. The purpose of this study was to quantitatively evaluate changes in taste before and after RT using IMRT or IMPT in patients with HNC, and to examine the potential usefulness of these findings in future clinical practice.

## MATERIALS AND METHODS

This retrospective study was approved by the Clinical Research Administration Center at our hospital and conducted in accordance with Guideline II of the Helsinki Declaration. The study involved 18 patients with malignant lesions of the head and neck region (15 men and 3 women; median age, 61 years; range, 45–76 years) who were treated at our hospital between October 2018 and July 2022. The patients were diagnosed with squamous cell carcinoma of the nasopharynx (*n* = 6), oropharynx (*n* = 7), or hypopharynx (*n* = 2); poorly differentiated carcinoma of the nasopharynx (*n* = 2); or carcinoma of the parotid gland (*n* = 1). All patients received IMRT or IMPT either as inpatients or outpatients, 5 times per week, at 70 Gy in 35 fractions over 7–8 weeks, or 65 Gy in 26 fractions over 6 weeks. In the dose constraints, the dose to the oral cavity was kept as low as reasonably achievable. The oral mean dose was 31.3 Gy for all head and neck patients, 21.8 Gy for IMPT patients, and 40.8 Gy for IMRT patients. The inclusion criteria were an age of ≧20 years, a confirmed pathological diagnosis of HNC, treatment with IMRT or IMPT, completion of both EGM measurements and patient-reported taste questionnaires, no refusal to participate in the study, and availability of complete EGM data at all predefined time points up to 12 months after completion of RT. We examined two sites on the tongue for each patient, measuring a total of 36 sites across the 18 study participants; however, two sites were excluded from the analysis, resulting in a total of 34 sites. One site was excluded because of prior surgical intervention and another site on the affected side of a patient with parotid gland cancer was excluded. Clinical demographic data and tumor stage distribution, based on the 8th edition of the Union for International Cancer Control (UICC) classification, are summarized in Table 1.

**Table 1:** Patient characteristics

Variables	Category	*n*	%
Age	Mean and range	61(45–76)	
Sex	Male	15	83.3
	Female	3	16.7
Radiation type	IMRT	9	50
	IMPT	9	50
Total dose of Gy	70Gy/35Fr	17	94.4
	65Gy/26Fr	1	5.6
Tumor site	nasopharynx	8	44
	oropharynx	7	39
	hypopharynx	2	11
	parotid gland	1	6
Tumor stage (UICC)	I	4	22
	II	4	22
	III	4	22
	IVA	5	28
	IVB	1	6

Abbreviations: IMRT: intensity-modulated radiotherapy; IMPT: intensity-modulated proton therapy

The patients were evaluated at five time points during RT: BL = baseline (before or within the first 5 days of RT initiation), CP = completion of RT, 3 M = 3 months after completion of RT, 6 M = 6 months after completion of RT, and 12 M = 12 months after completion of RT.

Regarding data completeness, EGM measurements were successfully obtained from all patients at all predefined time points. In contrast, patient-reported taste questionnaires were not completed in two patients at CP due to the inability to take oral intake. The number of evaluable patients at each time point is summarized in Table 2.

**Table 2:** Patient characteristics and availability of taste assessments

No		Radiation type	Tumor site	Stage	Oral cavity dose	Taste questionnaire	EGM testing
1	1-Right	IMPT	nasopharynx	I	14 Gy	○	○
	1-Left					○	○
2	2-Right	IMPT	nasopharynx	III	11 Gy	○	○
	2-Left					○	○
3	3-Right	IMPT	nasopharynx	III	22 Gy	△	○
	3-Left					△	○
4	4-Right	IMPT	nasopharynx	IVA	14 Gy	○	○
	4-Left					○	○
5	5-Right	IMPT	nasopharynx	IVA	13 Gy	○	○
	5-Left					○	○
6	6-Right	IMPT	oropharynx	I	42 Gy	○	○
	6-Left					○	○
7	7-Right	IMPT	oropharynx	II	22 Gy	○	○
	7-Left					○	○
8	8-Right	IMPT	oropharynx	II	34 Gy	○	○
	8-Left					○	○
9	9-Left	IMPT	parotid gland	IVB	27 Gy	○	○
10	10-Right	IMRT	nasopharynx	1	28 Gy	○	○
	10-Left					○	○
11	11-Right	IMRT	nasopharynx	II	27 Gy	○	○
	11-Left					○	○
12	12-Right	IMRT	nasopharynx	III	44 Gy	○	○
	12-Left					○	○
13	13-Right	IMRT	oropharynx	I	51 Gy	○	○
	13-Left					○	○
14	14-Right	IMRT	oropharynx	III	40 Gy	○	○
	14-Left					○	○
15	15-Left	IMRT	oropharynx	IVA	49 Gy	○	○
16	16-Right	IMRT	oropharynx	IVA	62 Gy	○	○
	16-Left					○	○
17	17-Right	IMRT	hypopharynx	II	30 Gy	△	○
	17-Left					△	○
18	18-Right	IMRT	hypopharynx	IVA	40 Gy	○	○
	18-Left					○	○

Abbreviations: IMRT, intensity-modulated radiation therapy; IMPT, intensity-modulated proton therapy; EGM, electrogustometry

The table summarizes individual patient characteristics, including radiation type, tumor site, stage and oral cavity dose, along with the availability of taste assessments. For the taste questionnaire and EGM testing, ‘○’ indicates that evaluations were successfully performed at all five time points, while ‘△’ indicates that evaluation was not performed only at the completion of radiation therapy but was available at all other time points.

### EGM testing

EGM testing allows for easy quantitative measurement of taste sensitivity by applying an electric current to the tongue to produce an electrical gustatory sensation [12, 14, 15]. The stimulus current can be adjusted in 2-dB steps, ranging from 4 μA (−6 dB) to 400 μA (34 dB), to determine the threshold at which the patient perceives taste. The electrical stimulus was delivered using an EGM (TR-06A; Rion Co., Tokyo, Japan) equipped with a flat, circular steel probe 5 mm in diameter. Measurements were taken at two sites (one on each side of the tongue apex, approximately 2 cm lateral to the anterior midline). These areas are innervated by the chorda tympani nerve. The average of three consecutive measurements was used to determine the electrical threshold. All patients rinsed their mouths with water before testing. First, the right side was measured, and after the value was confirmed, the left side was measured separately. Two sites were measured from each patient. Initially, a 30-dB stimulus was applied to confirm that the patient could perceive the EGM sensation. Testing then began with the lowest stimulus level (−6 dB) and was gradually increased until the patient reported perceiving taste.

### Patient-reported outcome measure

The patients completed a taste questionnaire to assess subjective taste perception. They rated the severity of taste abnormalities using a numeric rating scale (NRS), where 0 = no taste abnormality and 10 = no taste at all. Additionally, the patients evaluated their ability to perceive sweet, salty, sour and bitter tastes using a Likert scale ranging from 0 = no taste abnormality to 5 = no taste at all.

### Statistical analysis

All statistical analyses were performed using SPSS Statistics for Mac, version 32.0.0.0 (IBM Corp., Armonk, NY, USA). These analyses were used to evaluate differences in taste measures (changes in EGM thresholds, overall taste, sweet, salty, sour and bitter) across the five time points: baseline, completion of RT, 3 months after RT, 6 months after RT, and 12 months after RT.

Patient-reported outcome measures, including overall taste and the four basic tastes (sweet, salty, sour and bitter), were evaluated using the Friedman test. When a significant difference was detected, post-hoc pairwise comparisons were performed using the Wilcoxon signed-rank test with Bonferroni correction for multiple comparisons.

A linear mixed-effects model was used as the primary analysis to evaluate longitudinal changes in EGM thresholds, including fixed effects for time, treatment group (IMRT vs IMPT), and their interaction (time × group), with patient as a random effect. As supplementary analyses, non-parametric tests were performed. Within-group longitudinal changes were assessed using the Friedman test, followed by post-hoc pairwise comparisons using the Wilcoxon signed-rank test with Bonferroni correction. In addition, patient-level analyses were conducted using averaged bilateral EGM values. If one side was excluded, the value from the remaining side was used. The changes from baseline at each time point were analyzed using the Wilcoxon rank-sum test between the IMRT and IMPT groups, with Bonferroni correction applied for multiple comparisons.

A *P*-value of < 0.05 was considered statistically significant.

## RESULTS

### Patient-reported outcome measure

For the patient-reported taste questionnaire, data were missing in two patients at CP because oral intake was not possible at that time point. For all taste parameters evaluated (NRS, sweet, salty, sour and bitter), the Friedman test revealed significant longitudinal changes across the five time points (*P* < 0.001). Post-hoc pairwise comparisons with Bonferroni correction revealed that taste scores were significantly worse at completion of RT compared to baseline, with gradual recovery observed at later time points (Fig. 1). Significant differences were observed between baseline and completion of RT, between baseline and 3 months after completion of RT, between completion of RT and 6 months after completion of RT, and between completion of RT and 12 months after completion of RT. Detailed results of pairwise comparisons, including Bonferroni-adjusted *P*-values, are presented in Table 3.

**Fig. 1:**
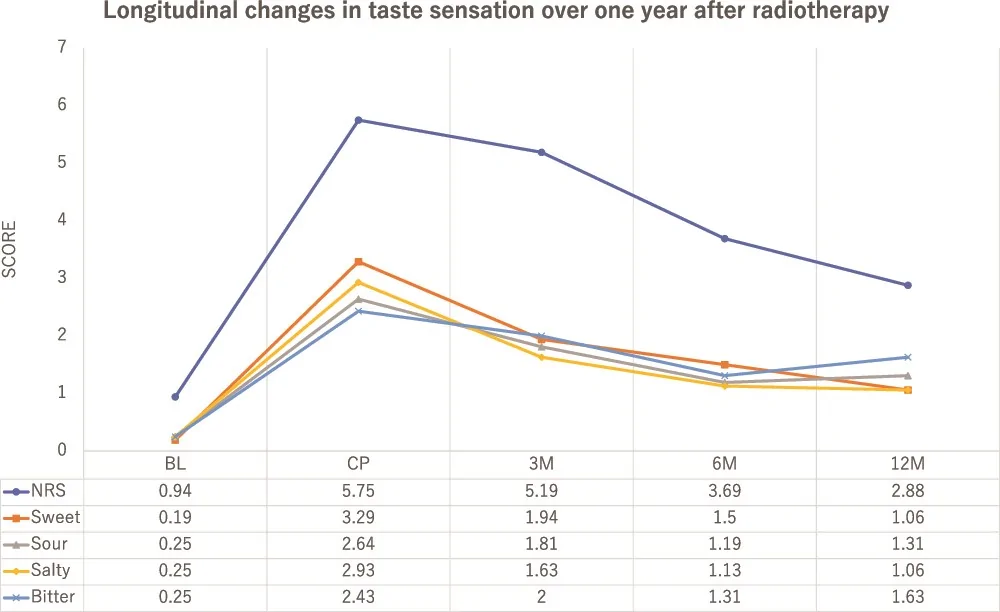
Longitudinal changes in taste sensation over one year after RT. Trends in taste sensation over one year. The x-axis indicates the evaluation time point: Baseline (BL, prior to RT), completion of RT (CP), 3 months after completion of RT (3 M), 6 months after completion of RT (6 M), and 12 months after completion of RT (12 M). Each graph represents a specific taste’s median value (NRS, sweet, sour, salty, bitter). The y-axis represents the average value of the Likert scale for each taste.

**Table 3:** Post-hoc pairwise comparisons of taste questionnaire across five time points using the Wilcoxon signed-rank test

	NRS	Sweet	Salty	Sour	Bitter
BL-CP	<0.001^**^ (<0.001^**^)	<0.001^**^ (<0.001^**^)	<0.001^**^ (<0.001^**^)	<0.001^**^ (<0.001^**^)	<0.001^**^ (0.003^**^)
BL-3 M	<0.001^**^ (<0.001^**^)	0.005^**^ (0.05)	0.015^*^ (0.153)	0.017^*^ (0.168)	0.002^**^ (0.023)
BL-6 M	0.034^*^ (0.336)	0.023^*^ (0.232)	0.073 (0.735)	0.12 (1)	0.107 (1)
BL-12 M	0.219 (1)	0.189 (1)	0.133 (1)	0.083 (0.831)	0.031 (0.314)
CP-3 M	0.78 (1)	0.12 (1)	0.094 (0.941)	0.073 (0.73)	0.55 (1)
CP-6 M	0.039^*^ (0.386)	0.036^*^ (0.365)	0.021^*^ (0.209)	0.009^**^ (0.086)	0.042^*^ (0.422)
CP-12 M	0.003^**^ (0.03^*^)	0.002^**^ (0.023^*^)	0.009^**^ (0.094)	0.014^*^ (0.143)	0.135 (1)
3 M–6 M	0.74 (0.736)	0.591 (1)	0.525 (1)	0.403 (1)	0.151 (1)
3 M–12 M	0.007^**^ (0.73)	0.135 (1)	0.356 (1)	0.511 (1)	0.37 (1)
6 M–12 M	0.371 (1)	0.339 (1)	0.773 (1)	0.858 (1)	0.591 (1)

Friedman test was used to evaluate differences in a taste questionnaire (NRS, sweet, salty, sour and bitter) across the five time points: baseline (BL, prior to RT), completion of RT (CP), 3 months after completion of RT (3 M), 6 months after completion of RT (6 M), and 12 months after completion of RT (12 M). Values represent *P*-values from the Wilcoxon signed-rank test for pairwise comparisons between time points. Bonferroni-adjusted *P*-values for multiple comparisons are shown in parentheses. ^*^P < 0.05, ^**^P < 0.01.

### EGM testing

All patients (*n* = 18) underwent EGM measurements at all time points without missing data. In 18% of head and neck RT patients, no change in the EGM threshold was observed, and no taste disorder occurred. Among this group, taste disorders were absent in 23% of IMPT patients and 11% of IMRT patients.

In the mixed-effects model, there was a significant effect of time on EGM thresholds (*P* < 0.001), indicating longitudinal changes following RT. However, neither the main effect of treatment group, such as IMPT and IMRT (*P* = 0.20), nor the interaction between time and treatment group (*P* = 0.40) was statistically significant.

In the site-level analysis, significant differences over time were observed in the overall cohort (*n* = 34), as well as in the IMPT group (*n* = 17) and IMRT group (*n* = 17) (all *P* < 0.001, Friedman test; Fig. 2). Post-hoc pairwise comparisons using the Wilcoxon signed-rank test with Bonferroni correction are presented in Table 4.

**Fig. 2:**
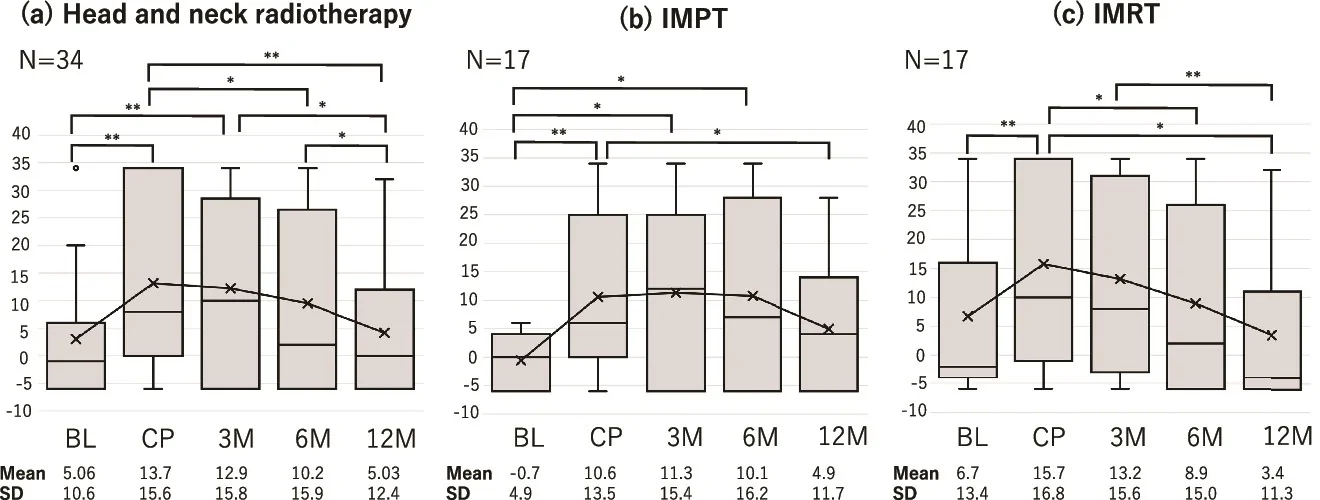
EGM thresholds over time: Box-and-whisker plots with mean profiles. Box-and-whisker plots with overlaid mean lines showing EGM thresholds at baseline (BL, prior to RT), completion of RT (CP), 3 months after completion of RT (3 M), 6 months after completion of RT (6 M), and 12 months after completion of RT (12 M) for (a) head and neck RT, (b) IMPT and (c) intensity-modulated radiotherapy (IMRT). Values are presented as median (interquartile range) for box plots, with mean values indicated by lines. SD, standard deviation. ^*^P < 0.05, ^**^P < 0.01.

**Table 4:** Post-hoc pairwise comparisons of EGM thresholds across five time points at the measurement-site level using the Wilcoxon signed-rank test

	Head and neck	IMPT	IMRT
BL-CP	<0.001^**^ (0.001^**^)	<0.001^**^ (0.008^**^)	0.026^*^ (0.262)
BL-3 M	0.003^**^ (0.032^*^)	0.013^*^ (0.126)	0.104 (1)
BL-6 M	0.076 (0.757)	0.017^*^ (0.17)	0.871 (1)
BL-12 M	0.85 (1)	0.329 (1)	0.193 (1)
CP-3 M	0.307 (1)	0.386 (1)	0.551 (1)
CP-6 M	0.028^*^ (0.284)	0.329 (1)	0.039^*^ (0.393)
CP-12 M	<0.001^**^ (<0.001^**^)	0.017^*^ (0.17)	<0.001^**^ (0.004^**^)
3 M–6 M	0.241 (1)	0.914 (1)	0.143 (1)
3 M–12 M	0.002^**^ (0.017^*^)	0.129 (1)	0.003^**^ (0.034^*^)
6 M–12 M	0.049^*^ (0.494)	0.159 (1)	0.143 (1)

Friedman test was used to evaluate differences in EGM testing among Head and neck, IMPT and IMPT patients across the five time points: baseline (BL, prior to RT), completion of RT (CP), 3 months after completion of RT (3 M), 6 months after completion of RT (6 M), and 12 months after completion of RT (12 M). Each measurement site was treated as an individual observation. Values represent *P*-values from the Wilcoxon signed-rank test for pairwise comparisons between time points based on site-level analysis. Bonferroni-adjusted *P*-values for multiple comparisons are shown in parentheses. ^*^P < 0.05, ^**^P < 0.01.

In the patient-level analysis, significant differences over time were also observed in the overall patient (*n* = 18), IMPT group (*n* = 9), and IMRT group (*n* = 9) (all *P* < 0.001, Friedman test). Post-hoc comparisons using the Wilcoxon signed-rank test with Bonferroni correction are summarized in Table 5.

**Table 5:** Post-hoc pairwise comparisons of EGM thresholds across five time points at the patient level using the Wilcoxon signed-rank test

	Head and neck	IMPT	IMRT
BL-CP	<0.001^**^ (0.004^**^)	0.017^*^ (0.171)	0.011^*^ (0.113)
BL-3 M	0.004^**^ (0.044^*^)	0.074 (0.736)	0.025^*^ (0.253)
BL-6 M	0.51 (0.512)	0.101 (1)	0.412 (1)
BL-12 M	0.874 (1)	0.881 (1)	0.709 (1)
CP-3 M	0.493 (1)	0.551 (1)	0.766 (1)
CP-6 M	0.114 (1)	0.456 (1)	0.086 (0.865)
CP-12 M	<0.001^**^ (0.002^**^)	0.025^*^ (0.253)	0.004^**^ (0.037^*^)
3 M–6 M	0.37 (1)	0.881 (1)	0.157 (1)
3 M–12 M	0.003^**^ (0.027^*^)	0.101 (1)	0.009^**^ (0.091)
6 M–12 M	0.35 (0.35)	0.136 (1)	0.233 (1)

Friedman test was used to evaluate differences in EGM testing among Head and neck, IMPT, and IMPT patients across the five time points: baseline (BL, prior to RT), completion of RT (CP), 3 months after completion of RT (3 M), 6 months after completion of RT (6 M), and 12 months after completion of RT (12 M). Patient-level values were calculated as the mean of bilateral measurement sites for each patient. Values represent *P*-values from the Wilcoxon signed-rank test for pairwise comparisons between time points based on patient-level analysis.

Bonferroni-adjusted *P*-values for multiple comparisons are shown in parentheses.

For the analysis of changes from baseline, comparisons between the IMRT and IMPT groups were performed using the Wilcoxon signed-rank test with Bonferroni correction. No significant difference was observed between BL-CP. For BL-3 M and BL-6 M, *P* = 0.15 suggested a tendency toward a significant difference. For BL-12 M, *P* = 0.04 indicated a significant difference (Fig. 3).

**Fig. 3:**
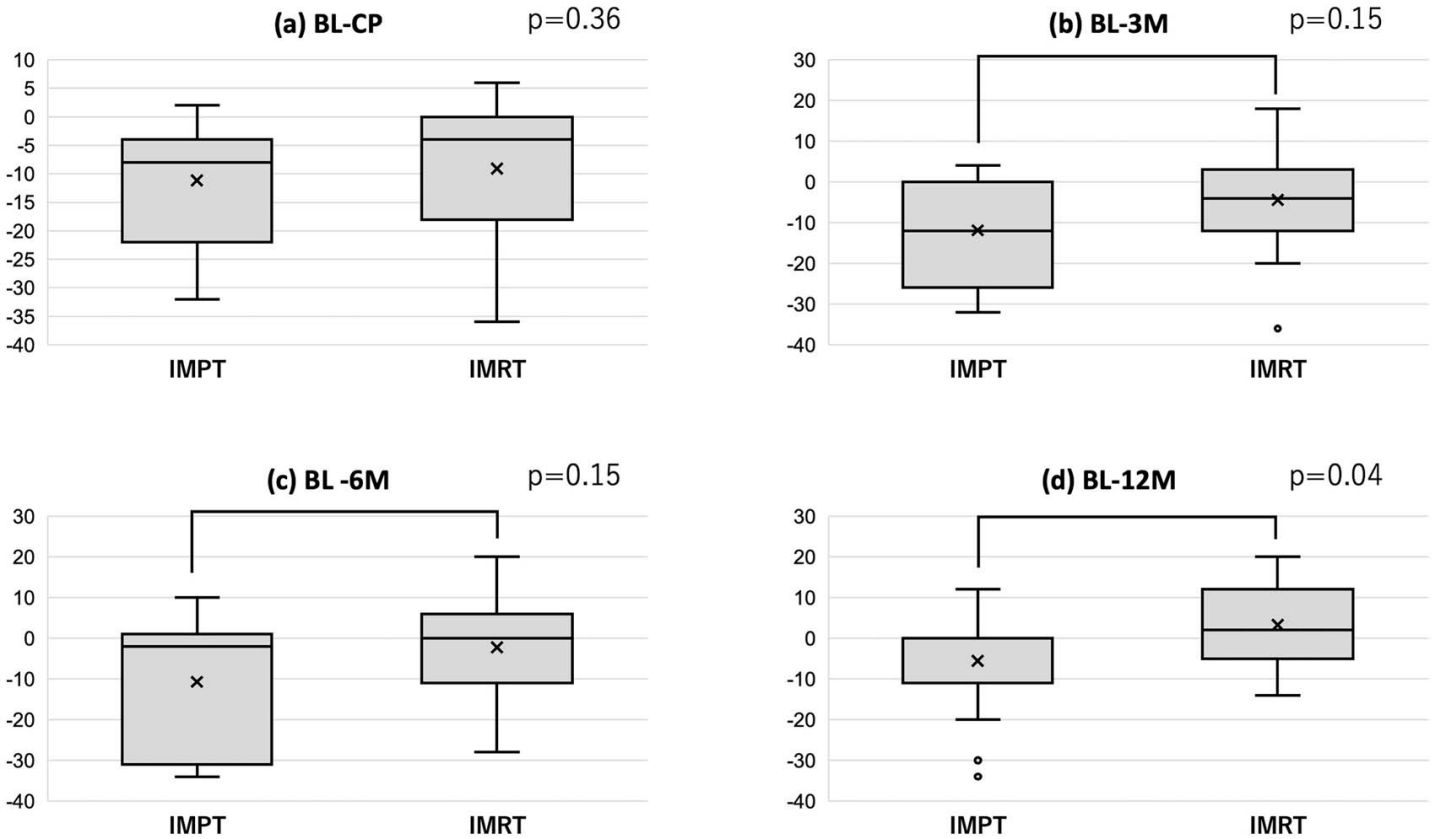
Changes from baseline at each time point: Comparison between IMPT and IMRT groups. Box-and-whisker plots showing changes from baseline at (a) BL-CP, completion of RT (CP), (b)BL-3 M, 3 months after completion of RT (3 M), (c) BL-6 M, 6 months after completion of RT (6 M) and (d) BL-12 M, 12 months after completion of RT (12 M), comparing the IMPT and IMRT groups.

In this study, no significant correlation was found between EGM threshold and radiation dose of oral cavity at any of the time points (CP, 3 M, 6 M, and 12 M) for all head and neck patients, IMRT patients and IMPT patients.

## DISCUSSION

Taste disorders are a common side effect in patients with HNC who undergo RT [2]. Evaluations of taste function often rely on subjective methods, such as patient-reported outcome measures [8, 9] and clinician-reported assessments [10, 11]. However, objective and straightforward evaluation methods capable of determining whether taste disorders have improved or resolved remain limited. Objective tests typically measure the minimum concentration of a tastant solution required for detection [13, 18]. By contrast, EGM provides a quantitative and reproducible means of assessing taste disorders caused by various factors, including Bell’s palsy, cancer and aging [14, 19]. EGM offers advantages such as a fixed measurement range, quantitative output and short testing time. In this study, we evaluated the clinical utility of EGM testing as an objective measure of taste function in patients with HNC treated with IMRT (17 sites) or IMPT (17 sites).

Patients undergoing head and neck RT were asked to rate their current sense of taste using a numerical scales (NRS). In patient-reported outcomes, adverse events associated with RT are generally assessed using an NRS. The ‘PRO-CTCAE’ system [20], which is a patient-reported outcomes tool developed by the US National Cancer Institute (NCI) to assess adverse effects of cancer treatment, also uses the NRS to evaluate the toxicity associated with radiation therapy. Based on NRS changes, the threshold rose sharply at CP, dropped slightly but remained elevated at 3 M, and decreased further toward baseline at 6 M. Many patients experienced taste disorders at the end of RT and for up to 3 months afterward. Previous studies have similarly reported that the prevalence of taste disorders peaks immediately after completion of RT [1, 7]. Subjective assessments have shown signs of recovery as early as a month post-RT, with near-complete recovery by 3 to 6 months [2, 4, 18]. The findings of this study are broadly consistent with those earlier reports.

In terms of changes in basic tastes (sweet, sour, salty and bitter), all tastes were most affected at CP and showed a recovery trend at 3 M. Pairwise comparisons suggested that sweet taste exhibited a greater number of significant differences across time points, whereas sour, salty and bitter tastes showed a similar number of significant differences. These findings may indicate that sweet taste tended to be more affected; however, the differences among the other tastes were not clearly distinguishable in the present analysis. Previous studies have typically reported that bitter and salty tastes are most affected, while sweet taste is least affected [6, 18, 21]. Another study found that sweet and bitter tastes were impaired after radiation exposure, whereas sour taste was not [22]. The reasons for these discrepancies remain unclear. However, because the present study relied on subjective evaluations based on patient self-reports, the findings should be interpreted with caution, and differences from objective taste test results are possible.

In the EGM testing of patients with HNC, the threshold increased most markedly at CP, showed a slight recovery trend at 3 M, and approached baseline levels at 6 M, indicating functional recovery. By 6 M, most patients exhibited taste recovery, though individual variability was observed and some still experienced residual disturbances. By 12 M, taste improvement was evident in nearly all patients. Previous reports using EGM testing have also shown elevated thresholds in all patients following conventional radiation therapy [14]. The results of the present EGM tests generally corresponded with subjective taste assessments using the NRS. Because EGM testing provides quantitative thresholds, it offers an objective method for evaluating taste function.

In this study, mixed-effects model demonstrated a significant effect of time but no significant differences between treatment groups or interaction effects. This suggests that EGM thresholds changed over time regardless of the treatment modality (IMPT or IMRT). In contrast, the supplementary analyses revealed significant temporal changes in both site-level and patient-level analyses. These findings may reflect increased sensitivity of non-parametric methods or differences in analytical units, particularly in the site-level analysis where measurements were treated as independent observations. Notably, the analysis of changes from baseline suggested a potential difference between IMRT and IMPT at 12 months, although no significant differences were observed at earlier time points. Given the lack of significant interaction in the mixed-effects model, this finding should be interpreted with caution and considered exploratory. IMPT using protons has been reported to reduce radiation exposure to the salivary glands and oral cavity—structures closely associated with taste function—more effectively than IMRT using X-rays [5, 17]. By selectively sparing these critical organs, IMPT may help mitigate the elevation of EGM thresholds. This potential advantage may partly explain the trend observed in the analysis at 12 M. Overall, while temporal changes in EGM thresholds were consistently observed, the evidence for differences between treatment modalities remains limited.

Several limitations of this study should be acknowledged. First, this was a retrospective study with a relatively small sample size. Although a larger number of patients were initially identified, only those who completed all EGM assessments up to 12 months after the completion of RT were included in the final analysis (*n* = 18). Patients who were lost to follow-up at 3 or 6 months—primarily due to death or refusal to continue examinations—were excluded from the analysis. Although patient dropout is not uncommon in HNC research, this selection process may have introduced bias into the study population. Second, this study did not account for the type, dose, or administration method of chemotherapy. Future studies should include a larger cohort, extend the follow-up period after irradiation, and incorporate additional variables that may influence outcomes, including the concomitant use of chemotherapy. Third, objective evaluation of basic tastes (sweet, sour, salty and bitter) was not performed. Future work should include chemical gustatory tests to determine the minimum concentration at which taste stimuli are detected or specific taste qualities are accurately identified, providing a more comprehensive objective assessment of taste function. Fourth, as a retrospective and non-randomized study, the comparison between IMPT and IMRT should be interpreted with caution. Potential confounding factors, including differences in oral cavity dose, use of chemotherapy, tumor site and treatment selection bias, may have influenced the results. These factors were not fully controlled in the present analysis. Therefore, the observed differences between the two treatment modalities cannot be attributed solely to the treatment effect. Future prospective, randomized studies with appropriate adjustment for confounding variables are warranted to validate our findings. Fifth, QOL scores such as the EORTC QLQ-C30 and QLQ-HN43 were not collected for the study participants. Comparing EGM results with QOL scores is expected to enhance the scientific rigor and clinical significance.

## CONCLUSIONS

In this exploratory study, gustatory dysfunction caused by RT was objectively evaluated using EGM testing in patients with HNC treated with IMRT or IMPT. A significant effect of time on EGM thresholds was observed, whereas no significant differences between treatment modalities were identified in the primary mixed-effects model. Although supplementary analyses suggested a tendency toward a more modest increase in EGM thresholds in patients treated with IMPT compared with those receiving IMRT, these findings should be interpreted with caution given the limited sample size and exploratory nature of the analyses. Therefore, while IMPT may have the potential to reduce radiation-induced taste dysfunction, further studies with larger and prospective cohorts are required to confirm these observations.

## Presentation at a conference

We presented this at the 66th Annual Meeting of Japanese Society for Oral and Maxillofacial Radiology on 30 and 31 May 2026.

## References

[1] Gunn L, Gilbert J, Nenclares P et al. Taste dysfunction following radiotherapy to the head and neck: a systematic review. Radiother Oncol 2021;157:130–40. 10.1016/j.radonc.2021.01.021. 33545253

[2] Asif M, Moore A, Yarom N et al. The effect of radiotherapy on taste sensation in head and neck cancer patients - a prospective study. Radiat Oncol 2020;15:144. 10.1186/s13014-020-01578-4. PMC7275377 32503643

[3] Chambers MS, Rosenthal DI, Weber RS. Radiation-induced xerostomia. Head Neck 2006;29:58–63. 10.1002/hed.20456. 16906511

[4] Sapir E, Tao Y, Feng F et al. Predictors of dysgeusia in patients with oropharyngeal cancer treated with chemotherapy and intensity modulated radiation therapy. Int J Radiat Oncol Biol Phys 2016;96:354–61. 10.1016/j.ijrobp.2016.05.011. 27473816

[5] Yasuda K, Minatogawa H, Dekura Y et al. Analysis of acute-phase toxicities of intensity-modulated proton therapy using a model-based approach in pharyngeal cancer patients. J Radiat Res 2021;62:329–37. 10.1093/jrr/rraa130. PMC7948838 33372202

[6] Maes A, Huygh I, Weltens C et al. De Gustibus: time scale of loss and recovery of tastes caused by radiotherapy. Radiother Oncol 2002;63:195–201. 10.1016/S0167-8140(02)00025-7. 12063009

[7] Ihara Y, Crary MA, Madhavan A et al. Dysphagia and oral morbidities in chemoradiation-treated head and neck cancer patients. Dysphagia 2018;33:739–48. 10.1007/s00455-018-9895-6. 29619560

[8] Fernando IN, Patel T, Billingham L et al. The effect of head and neck irradiation on taste dysfunction: a prospective study. Clin Oncol (R Coll Radiol) 1995;7:173–8. 10.1016/S0936-6555(05)80511-4. 7547520

[9] Jin S, Lu Q, Jin S et al. Relationship between subjective taste alteration and weight loss in head and neck cancer patients treated with radiotherapy: a longitudinal study. Eur J Oncol Nurs 2018;37:43–50. 10.1016/j.ejon.2018.10.003. 30473050

[10] Wise-Draper TM, Gulati S, Palackdharry S et al. Phase II clinical trial of neoadjuvant and adjuvant pembrolizumab in resectable local-regionally advanced head and neck squamous cell carcinoma. Clin Cancer Res 2022;28:1345–52. 10.1158/1078-0432.CCR-21-3351. PMC8976828 35338369

[11] Margalit DN, Schoenfeld JD, Rawal B et al. Patient-oriented toxicity endpoints after head and neck reirradiation with intensity modulated radiation therapy. Oral Oncol 2017;73:160–5. 10.1016/j.oraloncology.2017.08.012. 28939070

[12] Just T, Pau HW, Bombor I et al. Confocal microscopy of the peripheral gustatory system: comparison between healthy subjects and patients suffering from taste disorders during radiochemotherapy. Laryngoscope 2005;115:2178–82. 10.1097/01.MLG.0000181502.07160.86. 16369163

[13] Riva G, Raimondo L, Ravera M et al. Late sensorial alterations in different radiotherapy techniques for nasopharyngeal cancer. Chem Senses 2015;40:285–92. 10.1093/chemse/bjv011. 25800268

[14] Pavlidis P, Gouveris H, Gorgulla H et al. Electrogustometry and contact endoscopy findings in patients with head and neck malignancies treated with chemotherapy, radiotherapy, or radiochemotherapy. Chem Senses 2015;40:165–71. 10.1093/chemse/bju060. 25715386

[15] Pavlidis P, Gouveris H, Kekes G. Electrogustometry thresholds, tongue tip vascularization, density, and form of the fungiform papillae following smoking cessation. Chem Senses 2017;42:419–23. 10.1093/chemse/bjx009. 28379369

[16] Moreno AC, Frank SJ, Garden AS et al. Intensity modulated proton therapy (IMPT) - the future of IMRT for head and neck cancer. Oral Oncol 2019;88:66–74. 10.1016/j.oraloncology.2018.11.015. PMC6615027 30616799

[17] Sio TT, Lin HK, Shi Q et al. Intensity modulated proton therapy versus intensity modulated photon radiation therapy for oropharyngeal cancer: first comparative results of patient-reported outcomes. Int J Radiat Oncol Biol Phys 2016;95:1107–14. 10.1016/j.ijrobp.2016.02.044. PMC5409532 27354125

[18] Negi P, Kingsley PA, Thomas M et al. Pattern of gustatory impairment and its recovery after head and neck irradiation. Iran J Otorhinolaryngol 2017;29:319–27. PMC5785111 29383312

[19] Stillman JA, Morton RP, Hay KD et al. Electrogustometry: strengths, weaknesses, and clinical evidence of stimulus boundaries. Clin Otolaryngol Allied Sci 2003;28:406–10. 10.1046/j.1365-2273.2003.00729.x. 12969341

[20] Basch E, Reeve BB, Mitchell SA et al. Development of the National Cancer Institute's patient-reported outcomes version of the common terminology criteria for adverse events (PRO-CTCAE). J Natl Cancer Inst 2014;106:dju244. 10.1093/jnci/dju244. PMC4200059 25265940

[21] Baharvand M, ShoalehSaadi N, Barakian R et al. Taste alteration and impact on quality of life after head and neck radiotherapy. J Oral Pathol Med 2013;42:106–12. 10.1111/j.1600-0714.2012.01200.x. 22862149

[22] Barbosa da Silva JL, Doty RL, Miyazaki JVMK et al. Gustatory disturbances occur in patients with head and neck cancer who undergo radiotherapy not directed to the oral cavity. Oral Oncol 2019;95:115–9. 10.1016/j.oraloncology.2019.06.008. 31345378

